# Dasatinib reduces 5-Fu-triggered apoptosis in colon carcinoma by directly modulating Src-dependent caspase-9 phosphorylation

**DOI:** 10.1038/s41420-018-0062-5

**Published:** 2018-05-23

**Authors:** Yang Fu, Ge Yang, Peipei Xue, Luwei Guo, Yuhan Yin, Zhiqiang Ye, Sanfei Peng, Yanru Qin, Qiuhong Duan, Feng Zhu

**Affiliations:** 1 Department of General Surgery, The First Affiliated Hospital of Zhengzhou University, 450052 Zhengzhou, China; 2 Department of Ophthalmology, The First Affiliated Hospital of Zhengzhou University, 450052 Zhengzhou, China; 3 Department of Biochemistry and Molecular Biology, School of Basic Medicine, Huazhong University of Science and Technology, 430030 Wuhan, Hubei PR China; 4 Key Laboratory of Birth Defects and Reproductive Health of National Health and Family Planning Commission, Chongqing Population and Family Planning Science and Technology Research Institute, 400020 Chongqing, China; 5 Department of Obstetrics and Gynecology, The Third Affiliated Hospital of Zhengzhou University, 450052 Zhengzhou, China; 6 Department of General Surgery, Xinyang Central Hospital, 464000 Xinyang, China; 7 Department of Oncology, The First Affiliated Hospital of Zhengzhou University, 450052 Zhengzhou, China

## Abstract

Preclinical data have revealed the inhibitory effect of dasatinib on colon cancer. However, a combination of dasatinib and conventional chemotherapy has failed to show any meaningful outcome in a series of clinical trials. We, therefore, wondered whether Src kinase inhibitors were suitable for treating colon cancer in combination with chemotherapy drugs. This study was designed to explore whether dasatinib disturbed 5-Fu-triggered apoptosis in colon carcinoma. As a result, we established that Src was able to directly phosphorylate caspase-9 at tyrosine 251, leading to elevated caspase-9 activity. Dasatinib dramatically decreased 5-Fu triggered apoptosis in colon carcinoma via suppression of Src activation. Our findings may have partially explained why dasatinib combined with FOLFOX failed to show a meaningful clinical response in mCRC.

Colon cancer is a malignant disease that seriously threatens human health. It has been reported to be the third highest cause of cancer-related deaths^1^. Currently, surgical resection combined with fluorouracil-based chemotherapy is the main regimen for colon cancer. However, a part of patients are diagnosed with advanced or even metastatic colon cancer. These patients are burdened with a high relapse rate after surgery, while chemotherapy resistance ultimately leads to death. Therefore, it is imperative to explore molecular target therapy drugs, which can be used alone or combined with conventional chemotherapy, in order to overcome chemotherapy resistance in patients with metastatic colorectal cancer (mCRC).

c-Src protein, a member of Src family kinases encoded by Src gene, is the first proto-oncogene verified from the human genome^2^. Overexpression and overactivation of c-Src have been considered to be critically involved in carcinogenesis and cancer progression^3, 4^. Higher levels of Src kinase activity have been reported to contribute to the metastatic phenotype of colon cancer^5^. Dasatinib is an orally taken multi-target tyrosine kinase inhibitor that mainly targets c-Src and Bcr-Abl. At present, dasatinib is applied as a second-line drug in subjects with chronic myelogenous leukemia (CML) or Philadelphia chromosome-positive (Ph+) acute lymphoblastic leukemia (ALL)^6^. Despite the fact that some preclinical data^7–9^ have revealed the inhibitory effect of dasatinib against colon cancer, it remains unclear whether dasatinib can be used in metastatic colon cancer in clinical practice. In addition, dasatinib itself has been shown to exert no effect in previously treated mCRC patients^10^, and double suppression of epidermal growth factor receptor and c-Src by cetuximab and dasatinib, respectively, combined with FOLFOX did not show any meaningful clinical response in mCRC^11^.

Fluorouracil is able to cause DNA damage by altering the permeability of the outer mitochondrial membrane, releasing cytochrome-c and Smac to cytoplasm and formation of apoptotic bodies and aggregation and activation of caspase-9, which finally leads to caspase family-induced apoptosis^12^. Caspase-9 is a main initiating caspase member of endogenous apoptotic pathway^13^. There are many protein kinases regulating the activity of caspase-9 through phosphorylation^14^, such as extracellular signal–regulated kinase and CDK1, which phosphorylate caspase-9 at threonine 125 to inhibit caspase-9 activity^15, 16^. AKT phosphorylates caspase-9 at serine 196 to inhibit caspase-9 activity^17^. The only reported tyrosine protein kinase c-Abl can promote the activity of caspase-9 by phosphorylation of tyrosine 153^18^. Our previous study showed that Src kinase could directly phosphorylate as well as interact with caspase-7, leading to enhanced caspase-7 activity^19^.

Here, in this study, Src was found to directly phosphorylate caspase-9 at tyrosine 251, triggering an elevated caspase-9 activity. Dasatinib dramatically declined 5-Fluorouracil (5-Fu)-triggered apoptosis in colon carcinoma via inhibition of Src activation. Our finding may have partially explained why dasatinib combined with FOLFOX failed to show any meaningful clinical response in mCRC.

## Results

### Src directly phosphorylated caspase-9 at Y251

**Fig. 1:**
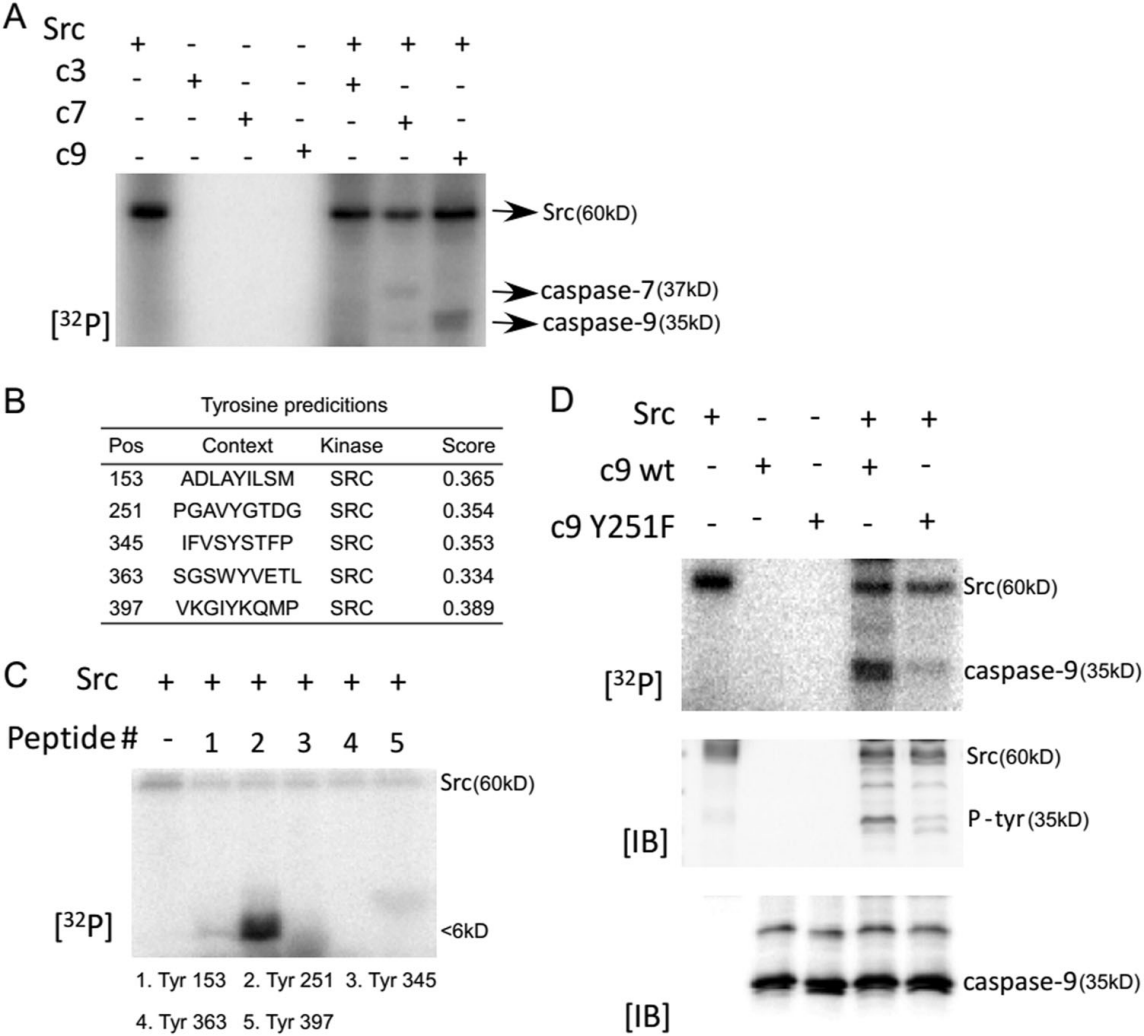
Src directly phosphorylate caspase-9 at tyrosine 251. **a** Src phosphorylate caspase-7 and caspase-9 in vitro. An in vitro kinase assay was conducted in the presence of [γ-^32^P] ATP. An equal amount (5 µg) of purified caspase-3, caspase-7, and caspase-9 proteins were loaded as potential substrates of Src active kinase (100 ng). **b** Use of NetPhos 3.1 program to predict possible tyrosine phosphorylation sites of caspase-9 by Src kinase. **c** Peptide mapping of five synthesized peptides containing each predicted tyrosine site. An in vitro kinase assay with Src active was conducted to examine each peptide. **d** Confirmation of tyrosine 251 phosphorylated by Src. Mutant caspase-9 Y251F protein was purified. Phosphorylation levels of Wt-caspase-9 or Mut-caspase-9 by Src in an in vitro kinase assay were visualized by autoradiography or by WB with p-tyrosine antibody. The same quantity of caspase-9 protein was assured by WB with caspase-9 antibody

We previously identified that Src could phosphorylate caspase-7 and promote 5-Fu-induced apoptosis^19^. Caspase-9 is an upstream initiator of caspase-3 and -7 in endogenous apoptotic pathway^20^. Hence, we speculated that Src may also phosphorylate caspase-9. To this end, an in vitro kinase assay was carried out with the existence of [γ-32P] ATP, where Src was utilized as an active kinase and equal amounts of caspase-3, -7, and -9 proteins were employed as substrates. Consequently, Src was able to phosphorylate caspase-9 in vitro. Moreover, a stronger phosphorylation signal was observed when Src interacted with caspase-9 other than with caspase-7 (Fig. 1a). NetPhos 3.1 software program was employed to predict the possible tyrosine phosphorylation sites of caspase-9 protein by Src kinase (Fig. 1b). After designing and synthesizing (PEPTIDE 2.0, Houston, TX, USA) five peptides, they were separately exposed to active Src in the presence of [γ-32P] ATP, which revealed that Y251 was strongly phosphorylated by Src (Fig. 1c). To further verify the outcomes from peptide mapping, mutant caspase-9 with Y251F was constructed utilizing the QuikChange Mutagenesis Kit. As a result, there was a dramatically reduced phosphorylation of caspase-9 Y251F protein by Src compared to Wt-caspase-9 either in the presence of [γ-32P] ATP or phosphor-tyrosine antibody (Fig. 1d), suggesting that Tyr251 was the most important phosphorylation site of caspase-9 by Src.

### Src increased caspase-9 activity in cells

**Fig. 2:**
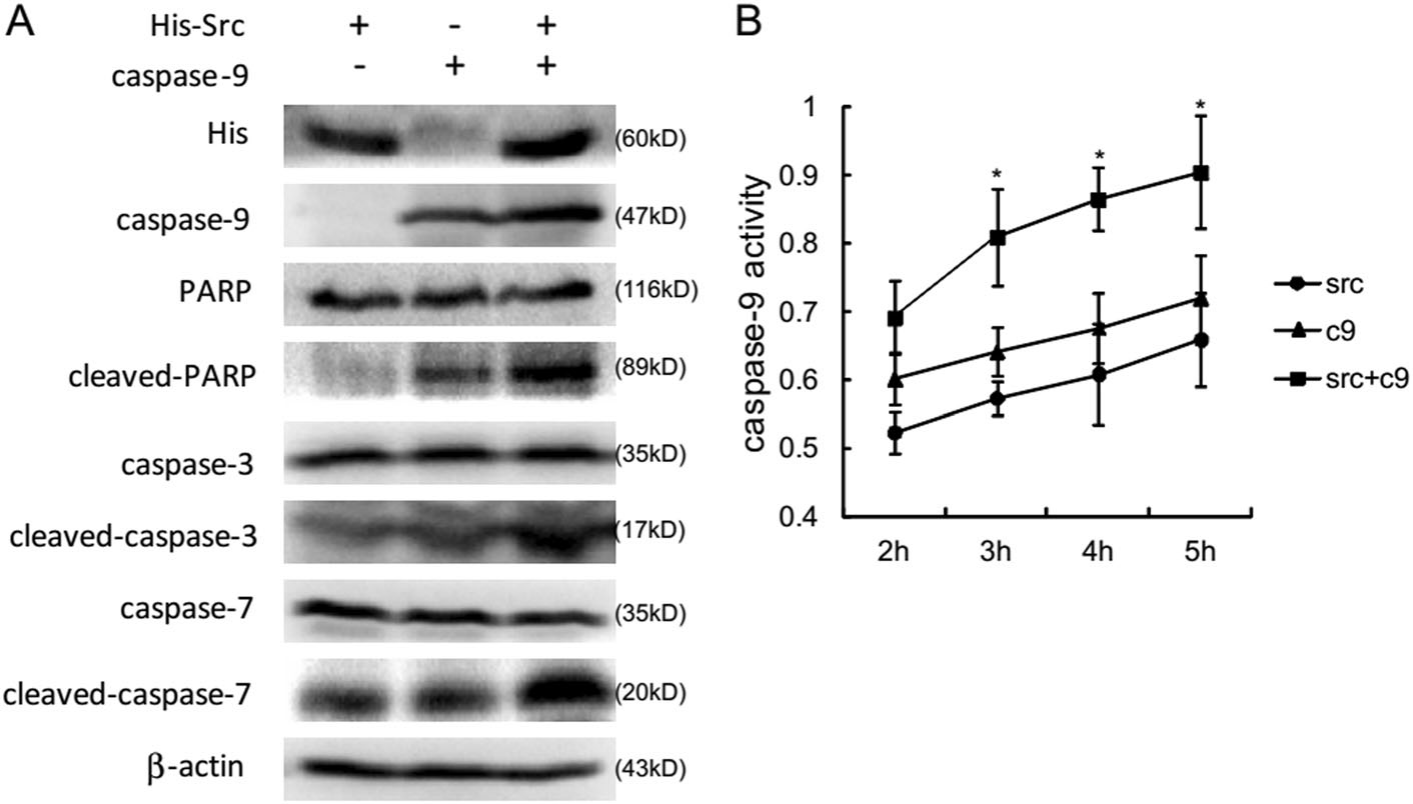
Src enhances caspase-9 activity in cells. **a** 293T cells were transfected with His-Src, caspase-9, or both. Cells were harvested 24 h after transfection. The expression level of several apoptotic proteins was detected by western blotting. **b** After transfection, 293T cell lysates were collected. Caspase-9 activity was measured using the Caspase-9 Colorimetric Assay kit. Data are shown as means ± S.D. of triplicate measurements. The asterisks indicate a significantly higher caspase-9 activity (*p *<* 0.05)

In order to assess the effect of Src phosphorylation exerted on caspase-9 activity, 293T cells were transfected with His-Src, caspase-9, or both, followed by evaluation of the apoptotic pathway using western blot (WB). Surprisingly, almost no endogenous caspase-9 was observed in 293T cells (Fig. 2a), which meant that 293T cells were perfect pure system to determine exogenous caspase-9 activity. More cleavage of poly ADP-ribose polymerase (PARP) as well as caspase-3 were detected in the caspase-9 alone group compared with the Src alone group. Dramatically increased cleavage of PARP, caspase-3 as well as caspase-7 were detected in the co-transfection group, indicating that Src enhanced caspase-9-related apoptotic pathway. Meanwhile, 293T cells transfected with His-Src, caspase-9, or both were harvested to test caspase-9 activity using the Caspase-9 Activity Kit. Consequently, co-transfection of Src and caspase-9 in 293T cells significantly increased caspase-9 activity in a time-dependent pattern (Fig. 2b). Together, the above findings implicated that Src may modulate apoptosis by increasing caspase-9 activity via phosphorylation.

### Dasatinib blocked 5-Fu-induced Src activation in colon carcinoma cells

**Fig. 3:**
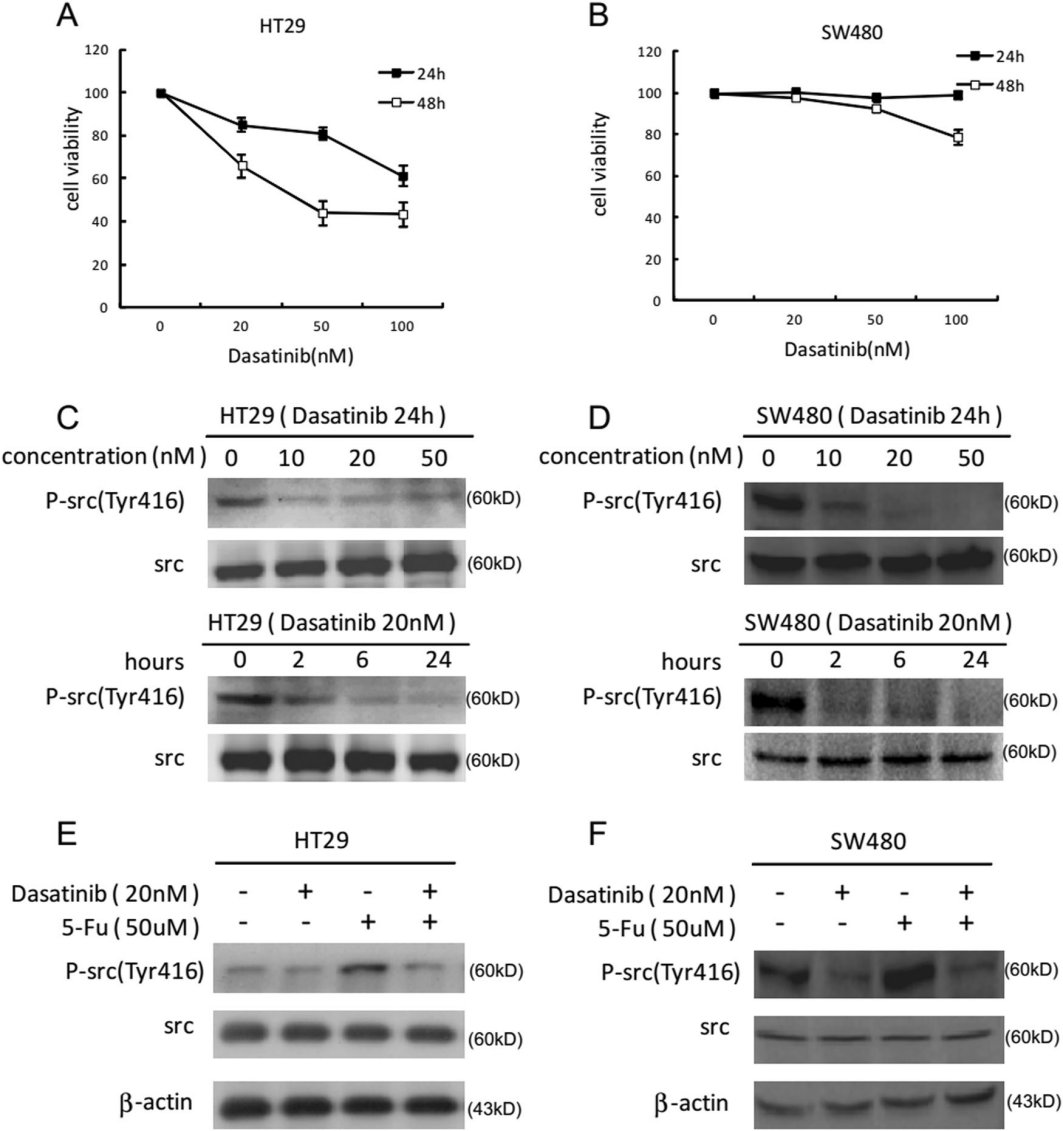
Dasatinib blocks 5-Fu-induced Src activation in colon cancer cells. An MTS assay was conducted to assess cytotoxicity at 24 and 48 h for HT29 (**a**) and SW480 (**b**) cells after being treated with dasatinib. Data are shown as the percentage of untreated control (100%) of triplicate measurements. Then the inhibitory effects of Src activity by dasatinib in HT29 (**c**) and SW480 (**d**) cells was detected using WB with p-src(tyr416). Next HT29 (**e**) or SW480 (**f**) cells were pretreated with dasatinib (20 nM) for 2 h and then incubated with 5-Fu (50 μM) for 48 h. The protein level of p-Src (Tyr416) and total src were probed by WB

We previously identified that 5-Fu could induce Src activation through generating ROS, and Src contributed to 5-Fu induced apoptosis^19^. Therefore, we hypothesized that dasatinib may disturb 5-Fu induced apoptosis by blocking Src activation. However, the previous study has suggested that high dose of dasatinib exerts synergistic effects with oxaliplatin in colon carcinoma cells^9^. Hence, we tried to detect a favorable dose of dasatinib that dramatically blocked Src activity but did not inhibit cell viability. An MTS assay was performed to detect cytotoxicity of dasatinib in HT29 and SW480 cells. As a result, HT29 cells exhibited dasatinib-induced cytotoxicity beyond 20 nM (Fig. 3a), while SW480 cells could tolerate dasatinib at 100 nM (Fig. 3b). Afterwards, WB was used to check the inhibitory effects of Src activity by dasatinib in HT29 and SW480 cells, indicating that dasatinib suppressed Src activity in a time- and concentration-dependent pattern in colon cancer cells. Incubation with 20 nM dasatinib for 2 h dramatically blocked Src activity in both HT29 and SW480 cells (Fig. 3c, d). Next, HT29 as well as SW480 cells were exposed to either dasatinib or 5-Fu alone or both for 48 h, demonstrating that 5-Fu administration triggered Src activation, which was further blocked by dasatinib (Fig. 3e, f).

### Dasatinib declined 5-Fu-triggered apoptosis in colon carcinoma cells

**Fig. 4:**
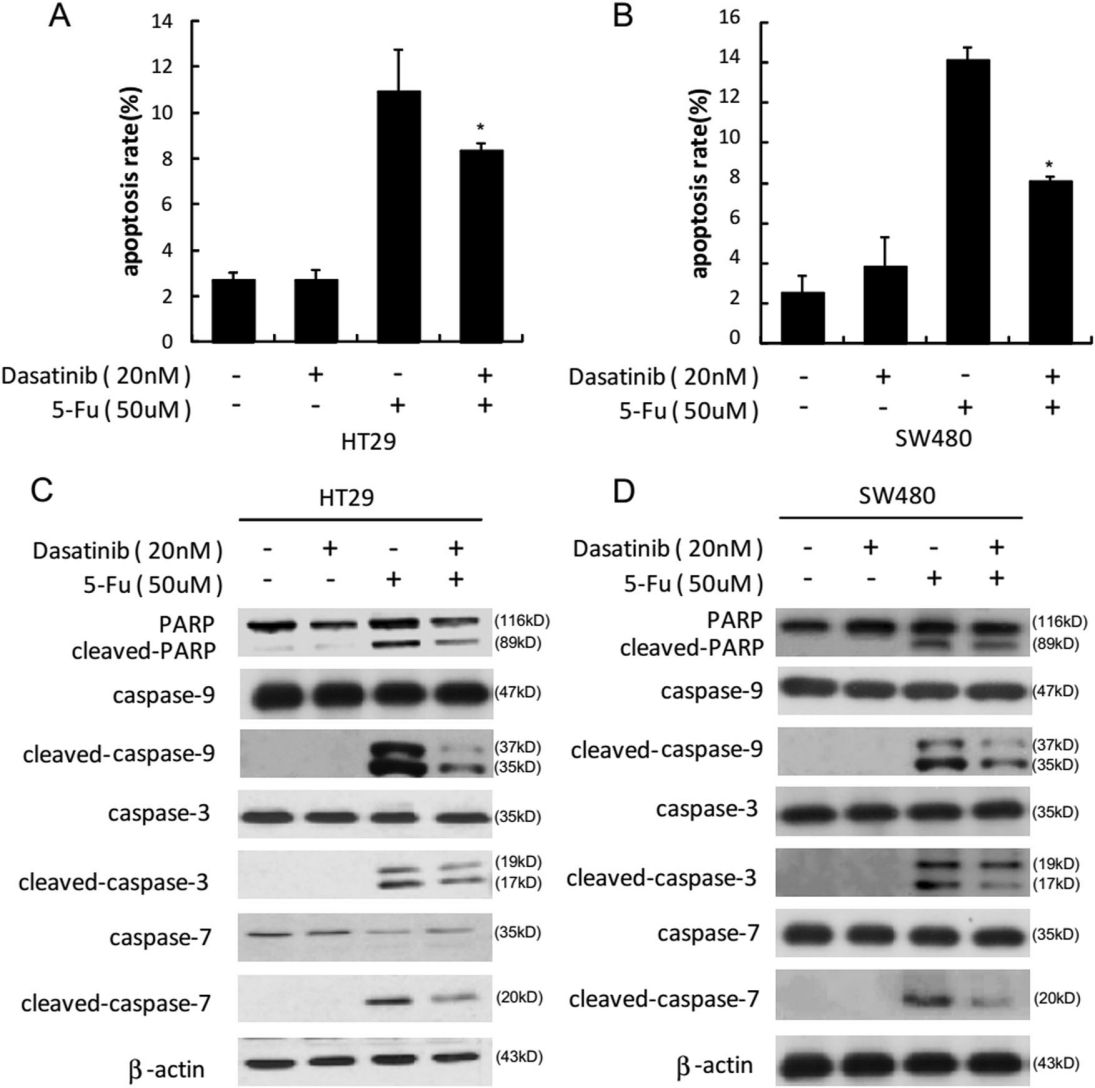
Dasatinib decrease 5-Fu-triggered apoptosis in colon carcinoma cells. HT29 or SW480 cells were pretreated with dasatinib (20 nM) for 2 h and then incubated with 5-Fu (50 μM) for 48 h. Early apoptosis was detected by flow cytometric analysis (**a**, **b**). Data were shown as means ± S.D. of triplicate measurements. The asterisks indicate a significant decrease in early apoptosis (*p *<* 0.05). Next HT29 or SW480 cells were treated as mentioned above and harvested. The protein level of total or cleaved PARP, caspase-3, caspase-7, and caspase-9 were probed by WB using specific antibodies (**c**, **d**)

In consideration that Src enhanced caspase-9 activity, and dasatinib blocked 5-Fu-triggered activation of Src, we then assessed whether a combination of dasatinib and 5-Fu could influence apoptosis in colon carcinoma cells. Consequently, 20 nM dasatinib (a dose that did not induce obvious cytotoxicity) declined 5-Fu-triggered apoptosis in HT29 (Fig. 4a) as well as SW480 cells (Fig. 4b), as indicated by flow cytometry. In addition, WB was performed to show that a combination of dasatinib and 5-Fu significantly reduced cleavage of caspase-3, -7, and -9 as well as PARP in HT29 (Fig. 4c) and SW480 cells (Fig. 4d) compared to administration with 5-Fu alone. Taken together, our findings suggested that dasatinib significantly reduced the apoptotic influences of 5-Fu in colon carcinoma cells.

## Discussion

Post-translational modifications of caspases, including glutathionylation, acetylation, phosphorylation, sumoylation, nitrosylation as well as ubiquitination are critically involved in regulation of apoptosis^21^. Caspase-9 is a main initiating caspase member of endogenous apoptosis. Intriguingly, tyrosine phosphorylation is able to enhance caspase-9 activity, while phosphorylation of multiple serine or threonine sites inhibits caspase-9 activity. Our previous study showed Src kinase could directly phosphorylate caspase-7, resulting in enhanced caspase-7 activity^19^. Our group purified several caspase proteins, including caspase-3, -7, and -9. A stronger phosphorylation signal was observed when Src interacted with caspase-9 rather than caspase-7. Of note, we proved that Tyr251 was the most vital phosphorylation site of caspase-9 by Src. Co-transfection of Src and caspase-9 in 293T cells significantly increased caspase-9 activity. Taken together, direct phosphorylation at Tyr251 by Src could enhance caspase-9 activity and subsequently regulate cell apoptosis.

Dasatinib is known as the only FDA-approved Bcr-Abl inhibitor used in patients with CML or Ph+ ALL who fail first-line therapy with imatinib. Since preclinical data showed the efficiency of dasatinib against multiple solid tumors including breast cancer^22, 23^, prostate cancer^24^, non-small-cell lung cancer^25^, and sarcoma^26^, many clinical trials of dasatinib in solid tumors either of monotherapy or in combination have been designed since 2010. Although in some phase I studies^27, 28^ dasatinib was associated with clinical benefits, it failed in almost all phase II^29^ and III^30^ studies either of monotherapy or in combination with chemotherapy or other target therapies. In consideration of Src overexpression in >80% of colon cancers, as well as dasatinib application in CRC cell lines supported by preclinical data, several clinical trials were also conducted in mCRC patients. However, dasatinib monotherapy was inactive in previously treated mCRC^10^. The combination of dasatinib plus FOLFOX also did not show a meaningful clinical activity in refractory CRC patients^11^. Similar results were observed in sunitinib (another multiple tyrosine kinase inhibitor that can inhibit Src) which failed in a phase III trial in combination with FOLFIRI in previously untreated mCRC^31^. We therefore wonder whether the Src kinase inhibitors are suitable for the combined therapy with conventional chemotherapy drugs in colon carcinoma.

The previous study has suggested that high doses of dasatinib have synergistic effects with oxaliplatin in colon cancer cells^9^. However, based on the phosphorylation results, we hypothesized that dasatinib should disturb 5-Fu-induced apoptosis. To explain the discrepancy, we conducted an MTS (3-(4,5-dimethylthiazol-2-yl)-5-(3-carboxymethoxyphenyl)-2-(4-sulfophenyl)-2H-tetrazolium) assay to detect cytotoxicity of dasatinib in colon carcinoma cells. The assay demonstrated that high doses of dasatinib exhibited cytotoxicity in some colon cancer cells. In this study, 20 nM dasatinib was chosen, which almost completely blocked Src activity without obvious cytotoxicity. As expected, dasatinib blocked 5-Fu-triggered activation of Src and significantly reduced the apoptosis influences of 5-Fu in colon carcinoma cells. Our results may have partially explained why dasatinib combined with FOLFOX failed to show any meaningful clinical response in mCRC.

## Materials and methods

### Reagents and antibodies

Dasatinib and 5-Fu as well as Src active kinase were commercially obtained from Sigma–Aldrich (St Louis, MO, USA) and Millipore Corp. (Billerica, MA, USA), respectively. Antibodies against total Src, ph-Src (Tyr416), caspase-3, PARP, cleaved caspase-3, and phospho-tyrosines as well as cleaved caspase-7 (Asp198) were obtained from Cell Signaling Technology (CST) (Boston, MA, USA). Antibodies against β-actin and caspase-7 as well as caspase-9 were obtained from Santa Cruz Biotechnology, Inc. (CA, USA).

### Cell culture and transfection

SW480, HT29, and HEK293T cells were purchased from ATCC and kept in line with the instructions by ATCC. All the cells were utilized within 6 months of resuscitation. SW480 and 293T as well as HT29 cells were maintained in Dulbecco’s modified Eagle’s medium and McCoy’s 5A medium (modified), respectively, containing 10% fetal bovine serum and antibiotics at 37 °C in a 5% CO_2_ humidified incubator. Simple-Fect was obtained from Signaling Dawn Biotech (Wuhan, China) for transfection. The transfection of pcDNA4-His-Src as well as pcDNA3-caspase-9 vector plasmids into 293T cells was performed at 50–60% confluence utilizing Simple-fect in line with the standard protocols.

### Purification of caspase-9 protein

Human pET-23b-His-caspase-9 was obtained from Addgene (Cambridge, MA), while caspase-9 mutants at Y251 (designated Y251F) were carried out with the QuikChange Mutagenesis Kit (Stratagene, Inc., La Jolla, CA, USA), followed by sequencing by Genewiz, Inc. The human caspase-3 as well as caspase-7 plasmids were obtained from Addgene, which were subsequently subcloned into the pET-46 Ek/LIC vector for purification. Afterwards, the induction of His-caspase-9-mutant as well as His-caspase-9-Wt proteins in *Escherichia coli* BL21 bacteria was carried out at 30 °C for 1 h by adding 0.5 mM isopropyl β-d-1-thiogalactopyranoside (IPTG). His-caspase-3 and His-caspase-7 were induced in *E. coli* BL21 bacteria and *E. coli* Rosetta (DE3), respectively, at 25 °C for 4 h by administration with 0.5 mM IPTG. Proteins purification was conducted utilizing nickel-nitrilotriacetic acid-agarose (Qiagen, Inc., Valencia, CA), followed by elution with 200 mM imidazole.

### In vitro kinase assay

Purified Wt-caspase-9, Mut-caspase-9, caspase-3, caspase-7, and caspase-9 peptides were exposed to active Src (Upstate Biotechnology, Inc., Boston, MA), 1 mCi of [γ-^32^P] ATP, 100 μM unlabeled ATP, as well as kinase buffer (CST). After incubation in a 32 °C waterbath for 40 min, 6× sodium dodecyl sulfate (SDS) loading buffer was employed to stop the reaction, followed by electrophoresis by SDS–polyacrylamide gel electrophoresis (PAGE) as well as visualization by autoradiography.

### Western blot analysis

The extraction of total protein was conducted using RIPA lysis with mixed protease inhibitors, followed by separation by SDS-PAGE and transferring to polyvinylidene difluoride membranes (Amersham Biosciences, Piscataway, NJ). Afterwards, the membranes were reacted with appropriate primary antibodies as well as subsequent horseradish peroxidase-conjugated secondary antibodies. The visualization of bands was carried out by an enhanced chemiluminescence reagent and exposure utilizing the ImageQuant LAS4000 system (GE, Piscataway, NJ).

### Caspase-9 activity assay

Caspase-9 Colorimetric Assay kit (Millipore Corp.) was purchased to assess caspase-9 activity in 293T cells. After collection of whole-cell lysates in each group, 200 µg of protein was utilized to assess caspase-9 activity in accordance with standard protocols.

### MTS assay

MTS Assay Kit (Promega, Madison, WI) was purchased to estimate cytotoxicity. Briefly, colon cancer cells were placed in 96-well plates (2000 cells per well), followed by incubation for 24 h. Then cells were exposed to the indicated concentrations of dasatinib, followed by incubation for another 24 or 48 h. After adding MTS, cells were incubated for 1 h at 37 °C in an incubator containing 5% CO_2_, followed by absorbance at a wavelength of 492 nm.

### Flow cytometric analysis

Annexin V-fluorescein isothiocyanate (FITC) Apoptosis Detection Kit (MBL International Corp., Woburn, MA) was purchased to conduct 5-Fu-triggered apoptosis in line with the manufacturer’s instructions. Briefly, cells were exposed to either 5-Fu or dasatinib or both, collected, washed with phosphate-buffered saline, as well as reacted with annexin V-FITC plus propidium iodide for 15 min at room temperature, followed by analysis by a FACS Calibur flow cytometer (BD Biosciences, San Jose, CA).

### Statistical analysis

All quantitative data were presented as mean ± standard deviation (SD). Statistical significance was assessed utilizing one-way analysis of variance. A *p*-value <0.05 was considered as statistically significant.
